# Missing elimination via membrane vesicle shedding contributes to the diminished calcium sensitivity of listeriolysin O

**DOI:** 10.1038/s41598-018-34031-4

**Published:** 2018-10-26

**Authors:** Jana Maurer, Sabrina Hupp, Helena Pillich, Timothy J. Mitchell, Trinad Chakraborty, Asparouh I. Iliev

**Affiliations:** 1 Institute of Anatomy, University of Bern, Baltzerstrasse 2, 3012 Bern, Switzerland; 2 Chair of Microbial Infection and Immunity, School of Immunity and Infection, College of Medical and Dental Sciences, University of Birmingham, Edgbaston, Birmingham, B15 2TT UK; 3 Institute of Medical Microbiology, Justus Liebig University Giessen, Schubertstr. 81, 35392 Giessen, Germany; 4 German Centre for Infection research (DZIF), Partner site Giessen-Marburg-Langen, Campus Giessen, Giessen, Germany; 5 Institute of Physiology and Pathophysiology, Heidelberg University, Im Neuenheimer Feld 326, 69120 Heidelberg, Germany; 6 DFG Membrane/Cytoskeleton Interaction Group, Institute of Pharmacology and Toxicology and Rudolf Virchow Center for Experimental Biomedical Science, University of Würzburg, Versbacherstr. 9, 97078 Würzburg, Germany

## Abstract

The lytic capacity of cholesterol-dependent cytolysins is enhanced in the extracellular calcium-free environment through a combination of limited membrane repair and diminished membrane toxin removal. For a typical neurotoxin of the group, pneumolysin, this effect has already been observed at reduced (1 mM) calcium conditions, which are pathophysiologically relevant. Here, we tested another neurotoxin of the group, listeriolysin O from *L*. *monocytogenes*, active in the primary vacuole after bacterium phagocytosis in host cells. Reduced calcium did not increase the lytic capacity of listeriolysin (in contrast to pneumolysin), while calcium-free conditions elevated it 2.5 times compared to 10 times for pneumolysin (at equivalent hemolytic capacities). To clarify these differences, we analyzed membrane vesicle shedding, known to be a calcium-dependent process for toxin removal from eukaryotic cell membranes. Both pneumolysin and listeriolysin initiated vesicle shedding, which was completely blocked by the lack of extracellular calcium. Lack of calcium, however, elevated the toxin load per a cell only for pneumolysin and not for listeriolysin. This result indicates that vesicle shedding does not play a role in the membrane removal of listeriolysin and outlines a major difference between it and other members of the CDC group. Furthermore, it provides new tools for studying membrane vesicle shedding.

## Introduction

Cholesterol-dependent cytolysins (CDCs) represent a large group of protein toxins that are produced by Gram-positive bacteria and contribute to the pathogenicity of their bacterial carriers^1^. Several common molecular characteristics are typical for these toxins: a cholesterol-specific recognition/binding motif (CBM), four molecular domains, and a highly homologous undecapeptide motif (ECTGLAWEWWR) positioned close to the CBM^1^. Members share a common mechanism of pore formation, which involves an initial alignment in oligomeric rings of 30 to 50 monomers along the membrane in the form of a prepore followed by unfolding of domain 3 and beta-hairpin formation to produce an aligned beta-barrel, thereby creating a hole in the cell membrane^2^. The mechanism of pore formation and membrane penetration by CDCs shares multiple similarities with the proteins of the perforin family. Therefore, in the literature, the collective term MACPF/CDC (Membrane Attack Complex/Perforin) superfamily of pore-forming proteins is used, as well^3^. Despite structural similarities, certain of these toxins demonstrate differences in cellular and organ tropism due to differences in the molecular structure and/or ecology of their hosts. Listeriolysin O (LLO), for example, is involved in the disruption of intracellular host vacuoles containing *Listeria monocytogenes*, facilitating pathogen escape and spread^4^, which is not characteristic of other members of the group.

LLO is studied largely in the context of its role in facilitating pathogen internalization, intracellular phagosome disruption, the release of *L*. *monocytogenes* and cell-to-cell spread of the pathogen^5^. LLO is the only member of the CDC group that is released by an intracellular pathogen and thus may affect host cell physiology both from inside and from outside the cell, although the relative contribution of each of these pools is still unknown^6^. Recently, we characterized the role of LLO as a neurotoxin, leading to dendritic changes in an NMDA-dependent manner when applied extracellularly but still differing from pneumolysin (PLY, produced by *Streptococcus pneumoniae*) (another well-studied neurotoxin of the same group) in its synaptotoxic activity^7^.

Calcium represents a critical factor in modulating the pore-forming capacity of many toxins, as it triggers multiple membrane repair and adaptive processes^8^. These processes include the endocytosis of damaged membrane fragments, membrane pore resealing and others^8,9^. Recent evidence suggests that cells utilize yet another calcium-dependent protective mechanism to counteract pore-forming toxins – the shedding of toxin-loaded extracellular vesicles^10^. Initially confirmed for PLY, the mechanism is now known to be relevant to other members of the CDC group, such as streptolysin, perfringolysin and intermedilysin^11^. Extracellular vesicle shedding is a calcium-dependent process; therefore, it is difficult to separate from local membrane repair processes^12^. A notable effect that can be explained by the diminished vesicle shedding after calcium depletion is the increased toxin membrane load and subsequently elevated cell lysis, studied in detail for PLY^13^. In this study, we addressed the question of whether LLO lytic capacity depends on calcium in a manner resembling other CDC toxins and especially PLY, as both toxins are neurotoxins, as well.

## Results

The experiments were performed in primary mixed mouse glial cells due to the neuropathological properties of LLO. First, the lytic capacity of our LLO preparation was analyzed and compared to another neurotoxin from the same group, PLY (see “Materials and Methods”). In all experiments, we utilized equivalent hemolytic amounts of LLO and PLY, as we focused on 2 HU/ml as the most relevant to the real disease concentration (for PLY with a lytic capacity of 40000 HU/mg, 2 HU/ml were equivalent to 0.2 µg/ml, which falls within the range of PLY in the cerebrospinal fluid of patients with pneumococcal meningitis^14^).

**Figure 1:**
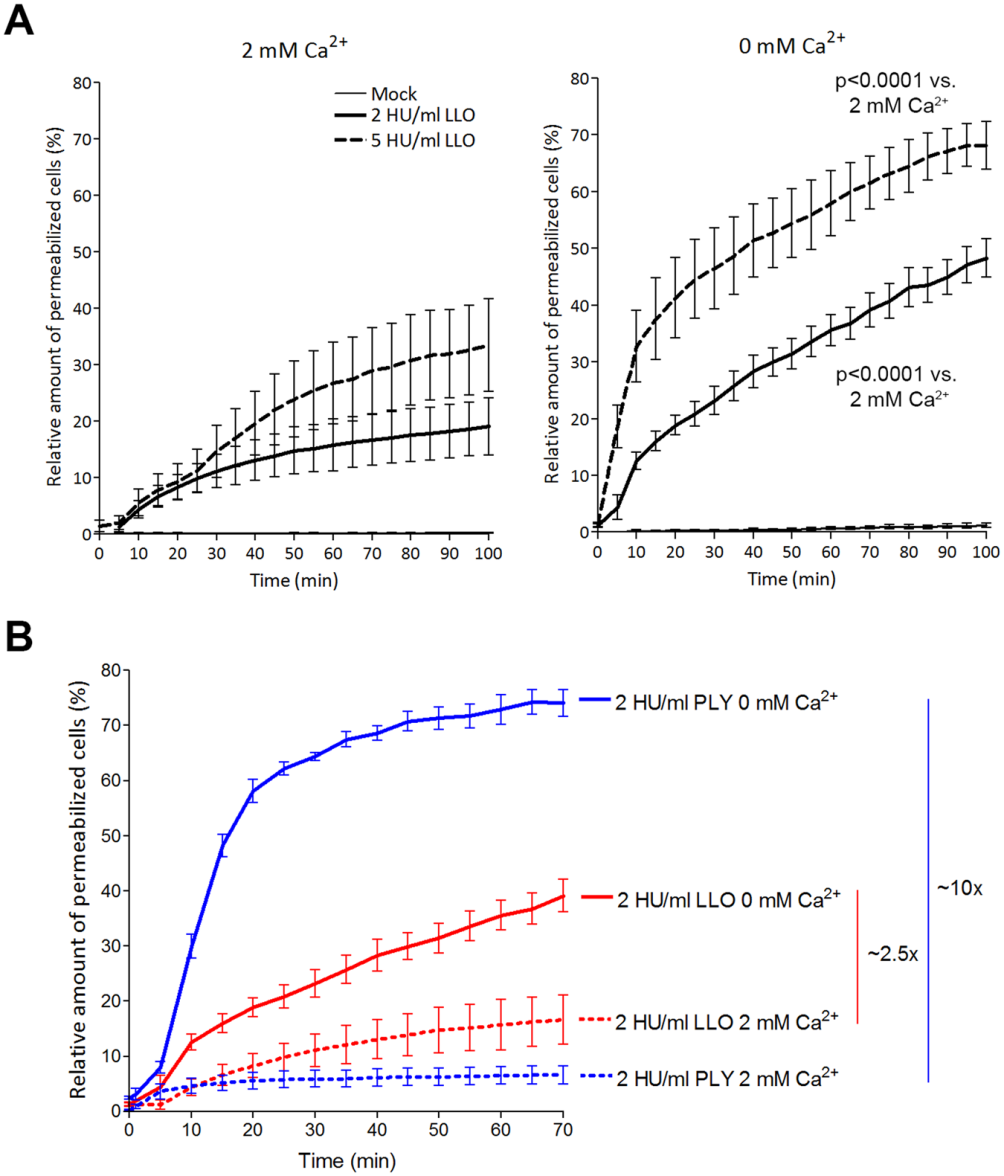
Cytolytic capacity of LLO and PLY in 2 mM Ca^2+^ and calcium-free conditions. (**A**) The lytic capacity (as judged by the fraction of propidium iodide-positive cells) of 2 and 5 HU/ml LLO increases with a reduction of calcium from 2 mM (left) to calcium-free conditions (right). Significance is calculated with the Wilcoxon matched pair test with individual time points paired to compare the lytic capacity of equivalent LLO concentrations at 0 and at 2 mM Ca^2+^. (**B**) Comparison of equivalent lytic amounts of LLO and PLY reveal much stronger increase after calcium removal for PLY (approx. 10 times) vs. LLO (approx. 2.5 times). All values represent the mean ± SEM of 5 independent experiments (n = 5).

Next, we evaluated cell permeabilization by 2 and 5 HU/ml LLO in both normal calcium (2 mM Ca) and calcium-free (0 mM Ca) conditions (Fig. 1) using a live imaging propidium iodide (PI)-based assay, where PI stains the nuclei of permeabilized cells. In calcium-free conditions, LLO’s lytic effect was enhanced in both the 2 and 5 HU/ml exposure groups (Fig. 1A). Compared with PLY (a known example of calcium dependence among CDCs) with equivalent hemolytic capacity, calcium depletion potentiated LLO lytic capacity less than that of PLY (approx. 2.5 times for LLO vs. 10 times for PLY; Fig. 1B).

**Figure 2:**
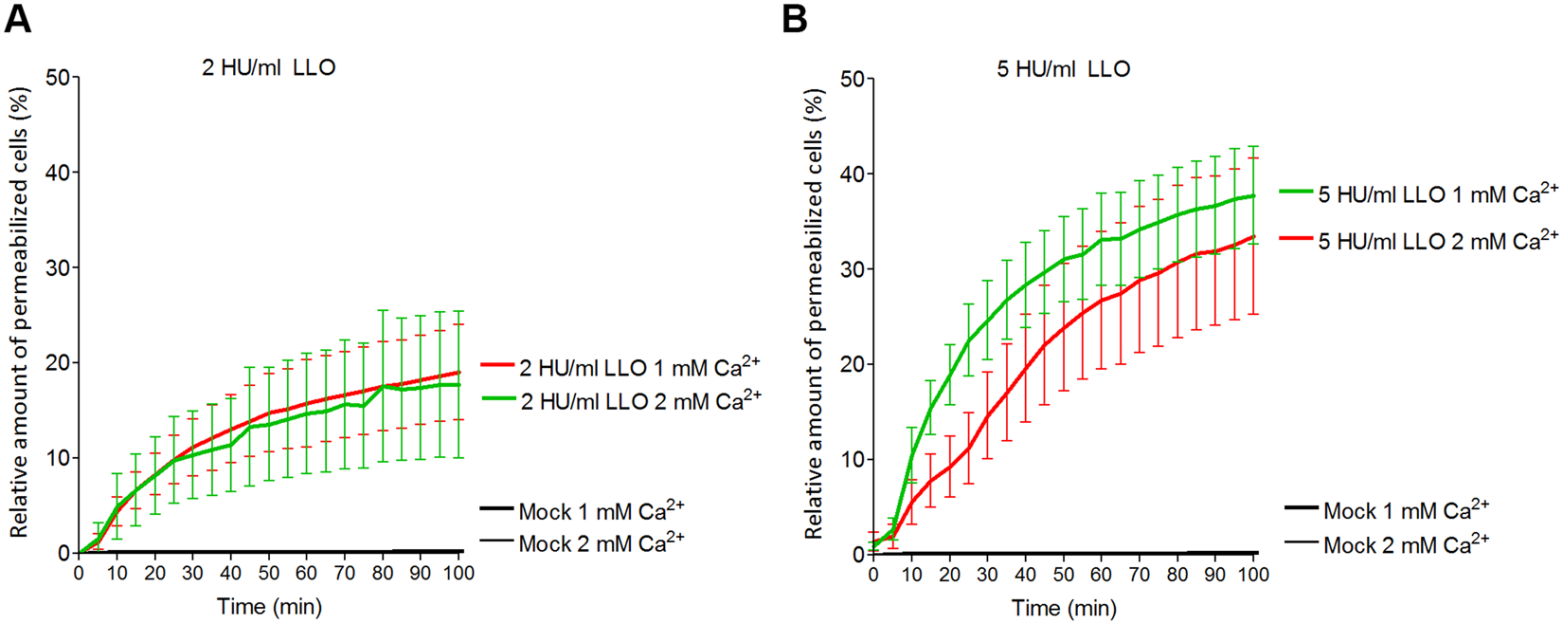
Cytolytic capacity of LLO in reduced calcium conditions. The reduction of calcium from 2 to 1 mM does not increase the lytic capacity of LLO when cells were challenged with 2 HU/ml (**A**) or 5 HU/ml (**B**). All values represent the mean ± SEM of 5 independent experiments (n = 5).

Calcium-free conditions represent an artificial system, which is not highly relevant to real disease pathogenesis. Decreased calcium concentration, however, is observed in some physiological and inflammatory conditions^15^. In the case of PLY, calcium reduction from 2 to 1 mM substantially increases toxin lytic capacity, which is important for such diseases as meningitis^13^. The lytic capacity of LLO, however, failed to increase when calcium was reduced to 1 mM both in the 2 HU/ml (Fig. 2A) and in the 5 HU/ml LLO (Fig. 2B) live imaging treatment groups.

**Figure 3:**
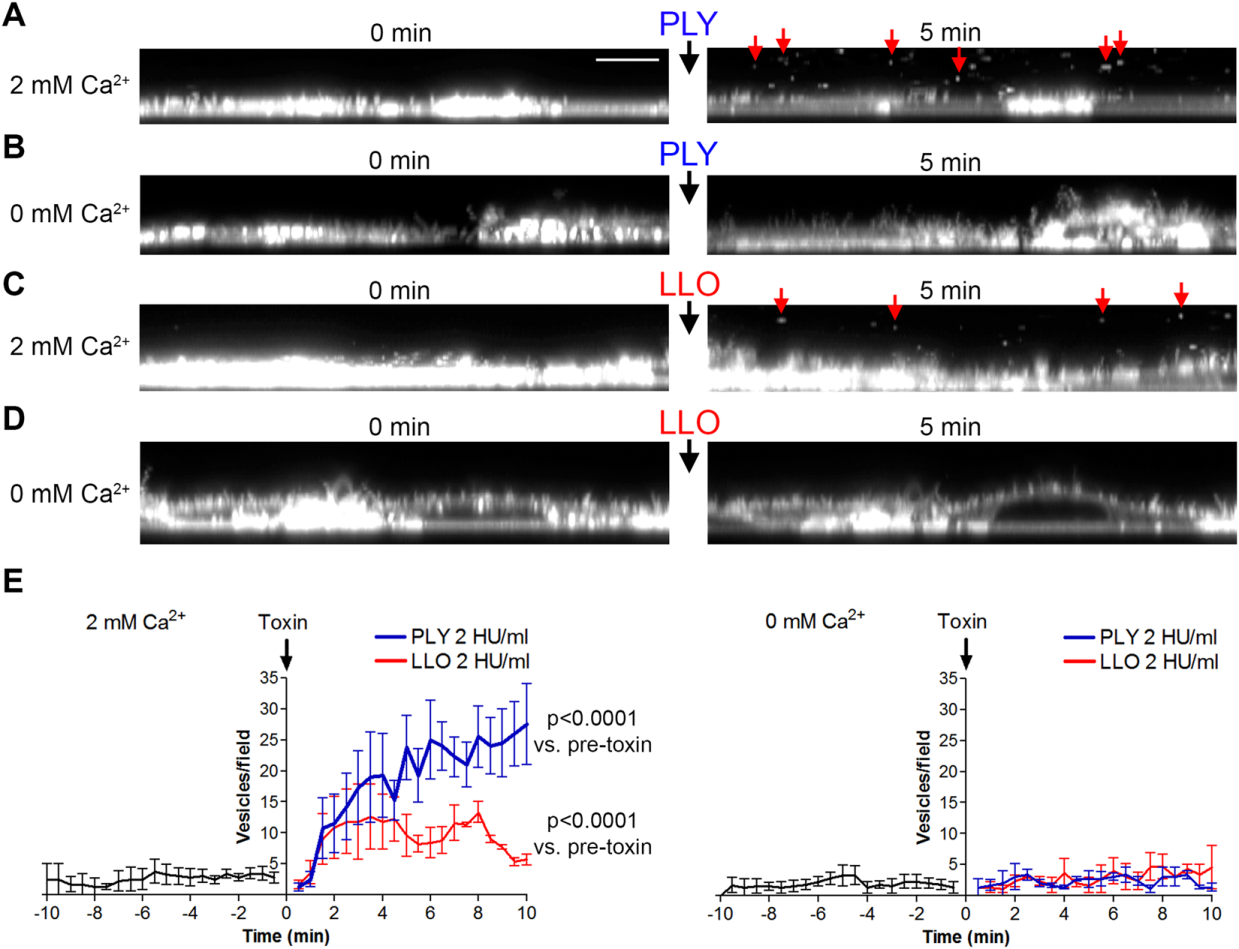
Extracellular vesicle shedding in primary mixed glial cells. Confocal imaging of fluorescent CellMask stained membranes (z-plane reconstruction in panels **A**–**D**) at 0 and at 5 min after exposure to toxin. (**A**) 2 HU/ml pneumolysin (PLY) in 2 mM Ca^2+^ conditions. Red arrows indicate shed extracellular vesicles. Scale bar: 8 µm. (**B**) Lack of membrane vesicle shedding after cell challenge with 2 HU/ml PLY in calcium-free conditions. (**C**) Membrane vesicle shedding (red arrows at 5 min) after challenge with 2 HU/ml listeriolysin (LLO). (**D**) Inhibited vesicle shedding after challenge with 2 HU/ml LLO. E. Diagrams of the number of vesicles/field (z-plane reconstruction field with the size 60 × 10 µm) before and after toxin challenge at 2 mM Ca^2+^ (left) and calcium-free conditions (right), clearly indicating the inhibition of vesicle shedding for both toxins in calcium-free conditions. All values represent the mean ± SEM of 5 independent experiments (n = 5). Significance calculated with the Wilcoxon matched pair test with individual time point pairing.

These experiments indicated the reduced calcium sensitivity of LLO vs. PLY. We know from previous research that the increased lytic effect of PLY in lower calcium conditions is due to increased membrane load as a result of the reduced vesicle toxin clearance by membrane vesicle shedding^10,13^. Therefore, we analyzed the ability of LLO to similarly induce calcium-dependent membrane vesicle shedding. We implemented a fluorescent vesicle staining protocol using CellMask Deep Red and high-speed live imaging laser scanning confocal microscopy^16^. As a positive control, we used PLY in equivalent lytic amounts. Indeed, PLY induced strong vesicle release in primary mixed glial cultures in concentrations of 2 HU/ml shortly after exposure (Fig. 3A,E, Supplementary Movie S1), which was completely blocked in calcium-free conditions (Fig. 3B,E, Supplementary Movie S2). Similarly, identical lytic amounts of LLO induced similar calcium-dependent shedding (Fig. 3C,E with calcium, Fig. 3D,E in calcium-free conditions; Supplementary Movies S3 (with calcium) and S4 (without calcium)). The peak of release, however, was lower and shorter when cells were treated with LLO versus PLY-treated cells (Fig. 3E).

**Figure 4:**
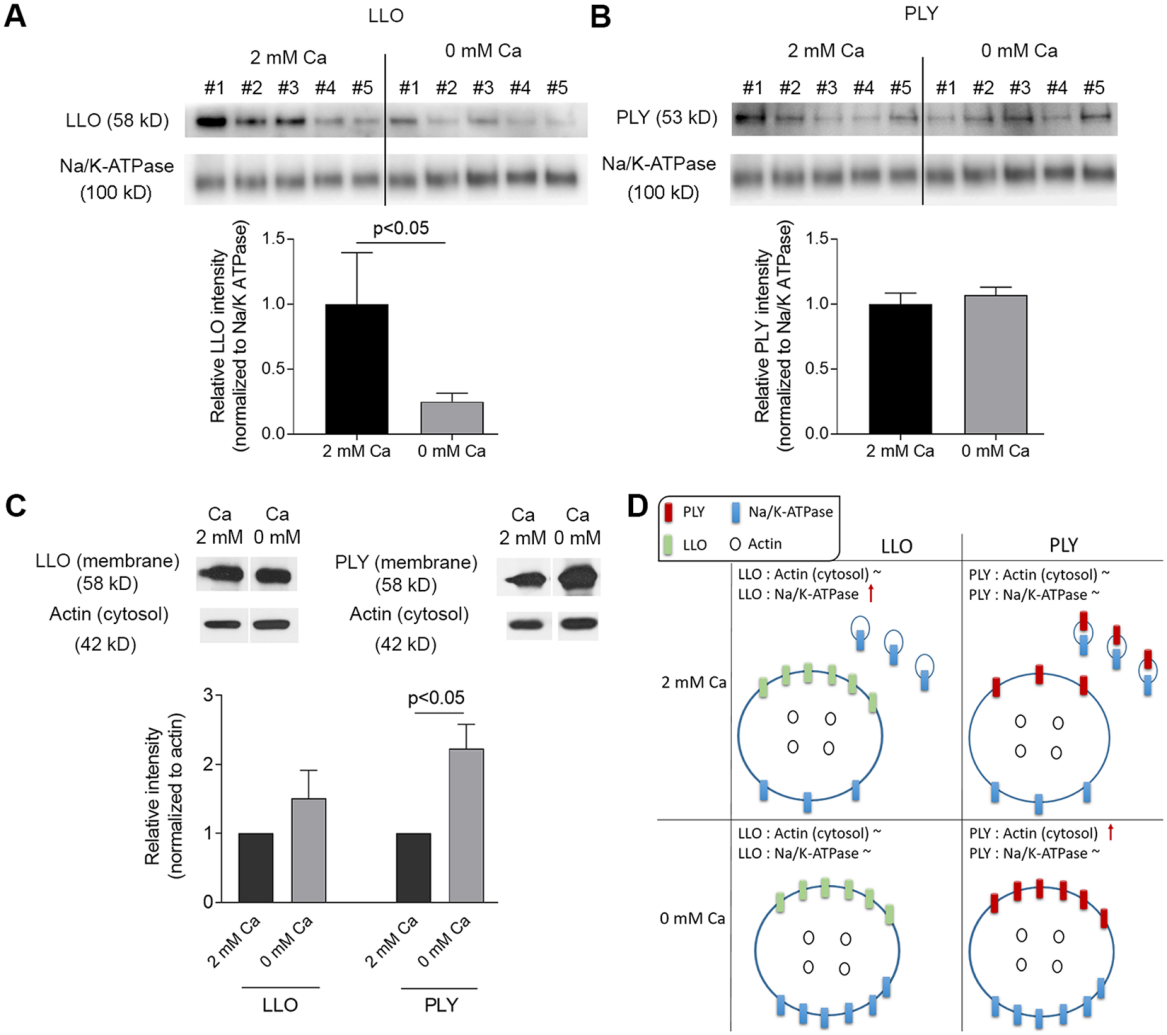
Western blot analysis of toxin load per membrane and per a cell. (**A**) Membrane-bound LLO after exposure to 2 HU/ml LLO for 20 min in 2 mM Ca^2+^ and in calcium-free conditions, normalized to the membrane marker Na/K-ATPase. The drop of LLO in calcium-free conditions indicates more Na/K-ATPase and less LLO is present in shed vesicles. #1 to #5 indicate independent experiments‘ samples. (**B**) Membrane-bound PLY after exposure to 2 HU/ml PLY for 20 min remains unchanged in 2 mM Ca2+ and in calcium-free conditions, normalized to the membrane marker Na/K-ATPase. This indicates shed vesicles contained proportional amounts of toxin and membrane marker. #1 to #5 indicate independent experiments‘ samples. (**C**) Membrane toxin load normalized to cytosolic actin (i.e. the number of cells) demonstrating increased load per a cell after calcium depletion for PLY, but not for LLO. (**D**) Schematic explanation of the results when normalizing to a membrane and to a cytosolic protein. All values represent mean ± SEM of 5 or 6 independent experiments (n = 5–6). All bands of interest are cropped from the source blots, as lines of cropping are indicated with dotted line. All loading controls with actin and Na/K-ATPase correspond to the identical samples with LLO or PLY staining in the same panel of the figure. Source blots in unprocessed form are included in Supplementary Figures S2–S4. Analysis is performed using the Wilcoxon matched pair test.

Next, we used Western blotting to analyze the amount of the membrane toxin load in crude membrane fractions after calcium depletion. We compared the toxin load in membranes normalized to the membrane marker Na/K-ATPase (Fig. 4A,B) and to the total cytosolic actin as a correlate for total cell number (Fig. 4C). We needed both controls due to the massive vesicle shedding after toxin challenge in 2 mM calcium, which depletes cell membranes and may be misleading alone. PLY remained unchanged per a relative membrane amount in normal calcium conditions (vesicles shed) and calcium-free conditions (no shed vesicles), indicating proportional loss of PLY and Na/K-ATPase (membrane amount marker) through vesicle shedding (Figs 4B and S2). LLO amount in total membranes was diminished when vesicles shedding was inhibited (calcium-free conditions), indicative for the disproportionately higher loss of Na/K-ATPase via shed vesicles as compared to LLO at conditions of 2 mM Ca (Figs 4A and S2). When comparing the total amount of toxin per a cell (Fig. 4C, cytosolic actin normalization, Supplementary Figs S3 and S4), LLO remained unchanged in normal calcium and calcium-free conditions, while the amount of PLY per a cell increased. Shortly, these experiments indicate that PLY initiated vesicle shedding with proportionate loss of PLY and membranes, while LLO initiated release of vesicles with less (if any) toxin in them.

## Discussion

In the current work, we outline the major differences between LLO and other typical member of the CDC group of toxins – PLY, regarding their calcium dependence and removal via membrane vesicle shedding. LLO activity was less dependent on calcium reduction, and it was not scavenged by shed membrane vesicles. The effect of reduced calcium concentration on toxin lytic capacity is of great importance due to its occurrence in relevant pathological conditions. Calcium concentration is reduced physiologically in the brain parenchyma of infant humans^17^ and in septic conditions^15^. For PLY, which was used for comparison throughout the work, the reduction of calcium from 2 to 1 mM (e.g., in newborn children) turns it from sub-lytic to lytic when exposing brain tissue to it^13^. For LLO, this effect was not observed.

The knowledge of the role of extracellular vesicles is growing very fast. It expands in the fields of tumor biology, infectious diseases, immunology and many other^18–20^. We still do not fully understand the biology of these vesicles – their generation, role, elimination^21^. Furthermore, we do not possess optimal experimental models to study them. Thus, understanding the mechanisms underlying toxin-induced extracellular membrane vesicle generation is of critical importance both as a biological phenomenon and as an accessible model system. Our results indicate that the concept of membrane repair by vesicle shedding in places of pores, although tempting with its logic and simplicity, may not hold true for all pore-forming toxins. Considering vesicle shedding as a pure membrane repair process should be carefully reassessed. On the other hand, CDCs are also used as model tools in other biology fields^22^, as cholesterol-binding probes^23^. Thus, understanding the differences in the membrane behavior of CDC toxins is also critically important for the interpretation of the experimental results, obtained with their help.

Various approaches for the visualization of membrane vesicle shedding exist – a number of laboratories focus on electron microscopy (EM) combined with biochemical isolation^10^, while others visualize microvesicles by fluorescent markers (GFP or fluorescent dyes)^16^. All of these approaches have advantages and disadvantages. EM analysis demonstrates a highly detailed picture, but only a fraction of all shed vesicles is visualized. The fluorescent labeling approach allows clear time resolution and visualizes all membrane phenomena, but fails to study the toxin load of individual vesicles. We decided to use the latter approach due to its real-time applicability and technical reproducibility. We combined it with the protein biochemistry of isolated membrane fractions to judge changes in total toxin load.

LLO is expressed and released from the food-borne pathogen *Listeria monocytogenes*^24^. *L*. *monocytogenes* is a pathogen that carries substantial public health relevance because of food poisoning risk, especially for immunosuppressed patients^25^. Another group of highly vulnerable patients are pregnant women, where listeriosis leads to stillbirths in 20% of cases^26^. Intracellularly, LLO is critical for the escape of *Listeria* from intracellular host vacuoles, allowing microorganisms to survive in the host cell and to spread to other neighboring cells^27^. The most serious complication of listeriosis is listerial meningitis and meningoencephalitis, where lethality reaches 70%^28^. We chose to use mixed glial cells (both astrocytes and microglia, as astrocytes represent 70–80% of all cells, and microglia represent 20–30%^29^), since both *L*. *monocytogenes* and *S*. *pneumoniae* often attack the brain^30,31^ and their pathogenic factors (in our case LLO and PLY) act on these cell types. The separation of microglia from astrocytes is possible, but the preparation of pure astrocyte cultures is highly challenging and practically difficult due to regular microglial contamination^32^. Furthermore, mixed cultures more closely resemble real tissues. Research in cell lines is informative, but may mechanistically differ from primary cells, in which infectious diseases normally develop. Therefore, we included both astrocytes and microglia in our analyses and did not observe any differences between them in their membrane vesicle shedding properties. The need for the comparison of LLO with another member of the CDC group required confirmation that the vesicle shedding effect of PLY, described in other cells (e.g., HEK293 cells), was present in our system, as well. Indeed, we confirmed the findings of other groups regarding PLY^33^ and extended them to primary cells.

As a major toxin of the pathogen, LLO is released either intra- or extracellularly^5^. The extracellular concentrations of LLO remain unclear, although multiple lines of evidence suggest a role of the extracellular toxin^6^. Experiments with acute brain slices demonstrate that LLO, applied at concentrations of 2 HU/ml and more, already causes dendritic changes in cortical neurons^7^. The specific role of LLO in disease cannot be explained only by its vacuole disruption effect. This role involves other likely extracellular roles, as demonstrated in experiments with *Listeria*, expressing perfringolysin O instead of LLO^34^. In this instance, perfringolysin-expressing mutants still escape from vacuoles effectively, but they fail to produce disease in animals. Evidence from other toxins of the group as well as from LLO confirms that CDCs disturb the functional fitness of cells in multiple ways.

In several earlier studies, the pH dependence of LLO effects and its loss of activity at physiological pH has been discussed^35^. While acidic conditions clearly enhance LLO’s lytic effect, in our hands, LLO preserved its lytic capacity at neutral pH long enough (at least a few hours) to allow effective interaction with host cells, especially in the brain^7^. Thus, defensive mechanisms of individual cells against extracellular LLO should be relevant to the disease course. Cell membranes are protected against pore-forming molecules by several complementary mechanisms – endocytosis of damaged membrane fragments^36^, exocytosis coupled with endocytosis^9^, exocytosis of lysosomes^37^ and “patching” of damaged membrane fragments (e.g., by annexin-A5)^8^. Virtually, all these mechanisms are calcium-dependent, which is also logical considering that calcium is a key second messenger for the cells and is strictly compartmentalized. Changes in calcium concentrations and its transmembrane fluxes indicate places of membrane damage. All described mechanisms of membrane repair have been considered as relevant to bacterial pore-forming toxins (many of them CDCs)^38^. Recently, the role of membrane vesicle shedding for protection against several pore-forming toxins (PLY^10^, perfringolysin, streptolysin and intermedilysin^11^) has been described. Membranes shed vesicles in various conditions – during apoptosis in the form of apoptotic blebs^16^, as a part of their physiological and/or pathological interaction with neighboring cells with the purpose of transferring receptors, messengers and cytosolic components^39,40^ or as a membrane repair tool^41^. These vesicles demonstrate different characteristics and sizes – exosomes (up to 200 nm) and microvesicles (up to 1 µm)^16^; a number of vesicles originate from the membrane per se^10^, while others are secreted as exosomes from multivesicular bodies from within the cells^42^. In all cases where extracellular vesicles have been released during challenge with pore-forming toxins, they carry toxin subunits and remove them from the plasmalemma to protect cells^9–11^. The final effect is that they remove membrane toxin, which is increased if vesicle shedding does not occur (e.g., in calcium-free conditions). Of course, other calcium-dependent membrane repair processes also contribute to plasmalemmal protection, but hardly any of them alter the effective membrane toxin load. Thus, the elimination of one of the calcium-dependent repair processes, vesicle shedding, from the total sum of calcium-dependent pore repair mechanisms should be logically considered as a factor contributing to the diminished calcium sensitivity of LLO. We cannot exclude, however, that other membrane repair processes, such as endocytosis and membrane resealing, are also differentially regulated in LLO compared to PLY.

The presence of calcium may influence the primary membrane binding of LLO and PLY differently. Bivalent ions can alter membrane fluidity, and theoretically, this may affect toxin binding as well^43^. Furthermore, some members of the CDC group demonstrate striking differences in their cholesterol-binding capacity, and they are able to discriminate different lipid environments^44^, i.e., they may bind different rafts. Such an effect should have been easily detectable by Western blot in our assays, but we did not see it. A direct effect of calcium on the pore-forming properties of the toxins (specifically for the calcium-dependent PLY) has been excluded in previous experiments with artificial membranes^13^.

When describing the increased membrane binding of PLY in calcium-free conditions^13^, we were not aware of the mechanistic role of vesicle shedding in membrane protection^10^. Logically, the increased membrane binding we observed there can be explained with reduced toxin elimination via the inhibition of membrane shedding as well. Once these vesicles remain membrane-bound, the number of membrane-bound toxin units rises. This effect was confirmed in the current work for PLY (as a positive control) and allowed for better demonstration of the contrast with LLO, namely, LLO induced vesicle shedding, as well, but its membrane load did not depend on the occurrence of these vesicles. With or without membrane vesicle shedding, the amount of LLO remained constant. In a broader perspective, this indicates that LLO is capable of “tricking” the cell to form extracellular vesicles while simultaneously escaping from them. One explanation for this finding is that LLO probably resides in different lipid microdomains than PLY, which are not directed towards extracellular vesicle shedding. While PLY resides in lighter sucrose gradient fractions, collocalizing with cholera toxin-B subunit (a lipid raft marker), listerilolysin was described to reside in heavier sucrose fractions^45,46^. Both works utilized different gradient conditions, protocols and cell membrane types, therefore no definitive conclusion can be made. We also lack knowledge about the exact membrane lipid microdomains sequestered by extracellular vesicles. Therefore, our findings represent a contribution to the limited set of tools to study these questions as well.

Another explanation of our findings may be that LLO is rapidly internalized before vesicle shedding occurs. A clue in this direction is the ability of LLO to enhance endocytosis, although PLY can produce similar effects^47^. These effects and the mechanism behind are still not well understood and further work in this direction is needed. Thus, we consider membrane shedding as a general stress response towards elevated calcium influx into the cell rather than a specific response towards all pore-forming toxins.

Indeed, the descriptive nature of our results overweights their mechanistic character. Unfortunately, calcium modulation represents one of the few experimental tools for extracellular vesicle modulation. Therefore, we believe our findings will provide additional tools in this challenging field.

The role of variations in calcium concentration as factors in toxin effects is just emerging. While PLY clearly benefits from mild calcium reduction, LLO remains largely insensitive to it. Differences among tissues and among conditions may have important implications for the progression of infectious diseases, creating largely permissive niches where specific pathogenic factors (such as CDCs) demonstrate variable effectiveness.

## Methods

### LLO and PLY preparation

LPS-free LLO was expressed and purified from the wild-type *L*. *innocua* 6a strain as described previously^48^. Briefly, overnight bacterial culture grown at 37 °C in BHI (brain-heart infusion) broth was used to inoculate the chemically defined minimal medium. Following 48 h incubation at 30 °C, bacteria were removed by centrifugation, and the supernatant was concentrated using a Millipore filtration apparatus with a cut-off point of 10 kDa. The crude supernatant of LLO was then batch-absorbed for with Q-sepharose or SP-sepharose (Pharmacia, Freiburg, Germany) and pre-equilibrated with loading buffer (50 mM NaH_2_PO_4_, pH 6.2). The non-absorbed fraction was centrifuged and desalted by transferring through a super loop to a HiPrep 26/10 desalting column (Pharmacia, Freiburg, Germany). Loading buffer (50 mM NaH_2_PO_4_, pH 6.2) was used to elute the desalted fraction. This fraction was subsequently filtered through a 0.22-μm filter and loaded onto a Resource-S column previously equilibrated with 50 mM NaH_2_PO_4_, pH 6.2. The pure toxin eluted reproducibly from the column at 0.21 to 0.28 M NaCl using elution buffer (50 mM NaH_2_PO_4_, 1 M NaCl, pH 5.6). Protein desalting and purification processes were carried out using the high-performance chromatography system ÄKTA explorer and UNICORN(tm) control system (Pharmacia, Freiburg, Germany).

Wild-type PLY was expressed in Escherichia coli BL-21 cells (Stratagene, Cambridge, UK) and purified via metal affinity chromatography. The purified PLY was tested for the presence of contaminating Gram-negative LPS using the colorimetric LAL assay (KQCL-BioWhittaker, Lonza, Basel, Switzerland). All purified proteins showed <0.6 endotoxin units/µg of protein. Hemolytic activity was judged on the basis of the standard assay described previosly^7^. Briefly, one hemolytic unit (HU) was defined as the minimum amount of toxin needed to lyse 90% of 1% human erythrocytes per ml within 1 h at 37 °C. Equivalent lytic capacity in red blood cells does not explicitly correspond to equivalent lytic capacity in other cell types^7^. For PLY, we determined hemolytic capacity of 40000 HU/mg and for LLO – 20000 HU/mg.

### Cell cultures and culture treatments

Primary mouse astrocytes were prepared from the cortices of newborn C57BL/6 mice (postnatal day (PD) 3–5) as mixed cultures with microglia in Dulbecco’s modified Eagle’s medium (high glutamate) (Thermo Fisher Scientific, Waltham, MA, USA). The growth medium was supplemented with 10% heat-inactivated fetal calf serum (FCS) (PAN Biotech GmbH, Aidenbach, Germany) and 1% penicillin/streptomycin (Thermo Fisher Scientific). Fourteen days after seeding in 75-cm^2^ cell culture flasks (Sarstedt AG & Co KG, Nuembrecht, Germany), the cells were harvested. Culture treatment with PLY and LLO was performed in serum-free medium.

All experiments involving animal materials followed the guidelines for ethical handling of experimental animals in Germany (Tierschutzgesetz, 2006) and in Switzerland (Tierschutzverordnung, 2008). The approvals for these experiments were granted by the Government of Lower Franconia (Würzburg, Germany) and the Cantonal Commission for Animal Experiments (Bern, Switzerland).

### Live imaging experiments

Cells were incubated in HEPES-based imaging buffer containing (in mM) NaCl 140, KCl 2, CaCl_2_ (2, 1 or 0), MgCl_2_ 1, HEPES 10, glucose 40 at pH 7.3^49^. Primary mouse glial cultures were visualized on an Olympus Cell^M imaging system (Olympus Deutschland GmbH, Hamburg, Germany) at 37 °C with the combination of a heating plate and custom-built microscope incubator with a heater and thermostat feedback loop using 10x and 20x dry objectives. PI and DAPI (4′,6-Diamidin-2-phenylindol) stains (Thermo Fisher Scientific) were used at end concentrations of 1 µg/ml. Vesicle shedding was analyzed on a Zeiss LSM 880 with Airyscan (Carl Zeiss AG, Oberkochen, Germany) using 63x oil immersion objectives and optical zoom between 2 and 4. Cell membranes were stained with CellMask Deep Red (Thermo Fisher Scientific) for 20 min at 1 µg/ml and visualized with a 633-nm laser line. Image processing and analysis were performed using ImageJ software (version 1.51 for Windows, National Institutes of Health, Bethesda, MD, USA).

### Protein biochemistry

Equal numbers of cells (500000 per 60 mm Petri dish) were washed with PBS and challenged either with LLO or PLY for 10–20 min at 37 °C in serum-free medium. Cells were washed three times with ice-cold PBS before proceeding further. Total crude cell membranes were isolated as described previously^7^. Shortly, cells were homogenized in buffer containing 10 mM Tris HCl (pH 7.4), 1 mM EDTA, 200 mM sucrose (all from Sigma-Aldrich Chemie GmbH, Munich, Germany) and protease inhibitor mix (Roche Diagnostics, Mannheim, Germany). The nuclei and cellular debris were removed by centrifugation at 900 × g for 10 min at 4 °C. The resulting supernatant was centrifuged at 110000 × g for 75 min at 4 °C. The crude membrane pellet was solubilized in buffer containing 10 mM Tris HCl (pH = 7.4), 1 mM EDTA, 0.5% Triton X-100 and protease inhibitor mix and boiled in Laemmli buffer at 95 °C for 20 min.

Samples containing equal amounts of protein (BCA test, Thermo Fisher Scientific) were loaded on a PVDF membrane (Schleicher & Schuell GmbH, Dassel, Germany) using a Novex® Tris-Glycine polyacrylamide gel system (Thermo Fisher Scientific). Precise protein adjustment was performed in experiments with cytosolic actin blot staining (marker for general cell number) and Na/K-ATPase blot staining (marker for membrane amount) by normalization to the respective loading control. After semi-dry blotting, the membranes were blocked with 3 or 5% non-fat milk and incubated with mouse anti-PLY antibody (ab71810, Abcam Plc, Cambridge, UK; 1:1000), rabbit anti-LLO antibody (ab200538, Abcam, 1:2000), mouse anti-actin antibody (Sigma-Aldrich; 1:2000) and anti-Na/K-ATPase antibody (Abcam, 1:15000). Cytosolic actin was used as a normalization loading control. After incubation with a horseradish peroxidase-conjugated goat anti-mouse and goat anti-rabbit secondary antibodies (Dianova, Hamburg, Germany), the bands were visualized using an ECL kit (GE Healthcare, Munich, Germany) and Kodak X-ray films, developer and fixative (Kodak, Rochester, USA). Films were scanned with professional HP Scanjet G4010 (Hewlett-Packard, Palo Alto, USA) and stored as 16-bit TIFFs. In some experiments, automatic imager Fusion FX (Vilber Lourmat, Collégien, France) was used. Analysis was performed in ImageJ, measuring the line profile of the bands (measurement along the width of bands) and calculating the area under the curve (thus integrating size and intensity of bands), considering the background as a baseline^50^. Furthermore, using standard concentration curve with recombinant toxin, we confirmed all samples remained within the linear range of evaluation (Supplementary Fig. S1). Representative blots are included in the figures; the complete sets of blot film scans are included as Supplementary Materials.

### Statistical analysis

Statistical analysis was performed using GraphPad Prism 4.02 for Windows (GraphPad Software Inc., La Jolla, CA, USA). The statistical tests included Mann-Whitney U tests (comparing two groups differing in one parameter) or Wilcoxon matched pairs test. Permeabilization kinetics analysis was performed by non-linear regression analysis utilizing one phase exponential association.

## Electronic supplementary material

Supplementary movie S1

Supplementary movie S2

Supplementary movie S3

Supplementary movie S4

Supplementary figures
