# Supplementary material for: Missing elimination via membrane vesicle shedding contributes to the diminished calcium sensitivity of listeriolysin O
Source: Sci Rep. 2018 Oct 26;8:15846. doi: 10.1038/s41598-018-34031-4 (PMC6203718; doi:10.1038/s41598-018-34031-4)

Missing elimination via membrane vesicle shedding contributes to the diminished calcium sensitivity of listeriolysin O

Jana Maurer, Sabrina Hupp, Helena Pillich, Timothy J. Mitchell, Trinad Chakraborty, Asparouh I. Iliev

SUPPLEMENTARY FIGURES

Supplementary figure S1. Analysis of Western blot-detected standard dilution curve of listeriolysin (LLO) indicating the accuracy of area/intensity integration (area x intensity) of the Western blot bands when intensity saturation is reached. Two linear regression modes were used – fitting all points and fitting only the 4 lower intensity points, both demonstrating excellent accuracy and linearity of the measurement values. Blue arrow indicates the highest detection level in our experimental Western blots, falling clearly within the linear range.

Supplementary figure S2. Unprocessed Western blot film scans of the LLO and PLY experiments in Figure 4A and 4B, including membrane loading Na/K-ATPase controls.

Supplementary figure S3. Unprocessed Western blot film scans of LLO experiments in Figure 4C including actin loading controls.

Supplementary figure S4. Unprocessed Western blot film scans of PLY experiments in Figure 4C including actin loading controls.

Supplementary figure S1. Analysis of Western blot-detected standard dilution curve of listeriolysin (LLO) indicating the accuracy of area/intensity integration (area x intensity) of the Western blot bands when intensity saturation is reached. Two linear regression modes were used – fitting all points and fitting only the 4 lower intensity points, both demonstrating excellent accuracy and linearity of the measurement values. Blue arrow indicates the highest detection level in our experimental Western blots, falling clearly within the linear range.

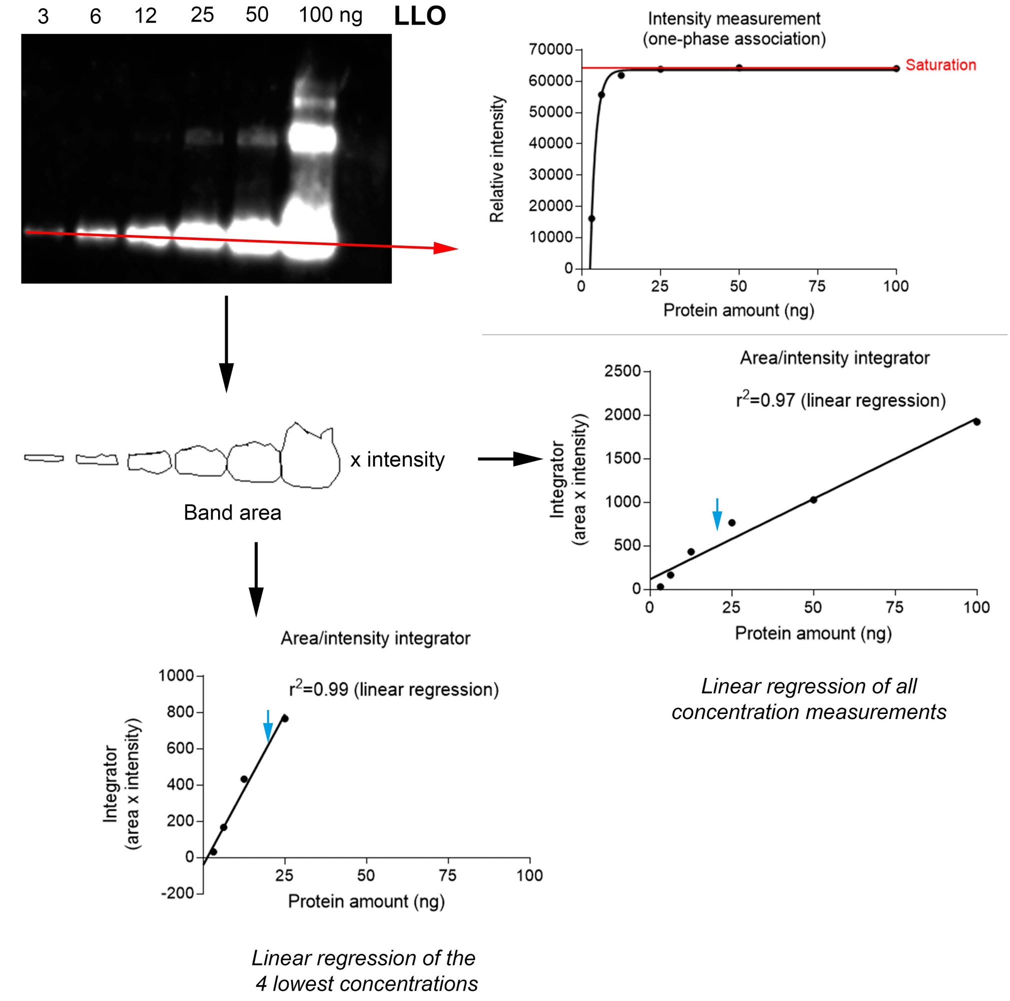

Supplementary figure S2. Unprocessed Western blot film scans of the LLO and PLY experiments in Figure 4A and 4B, including membrane loading Na/K-ATPase controls.

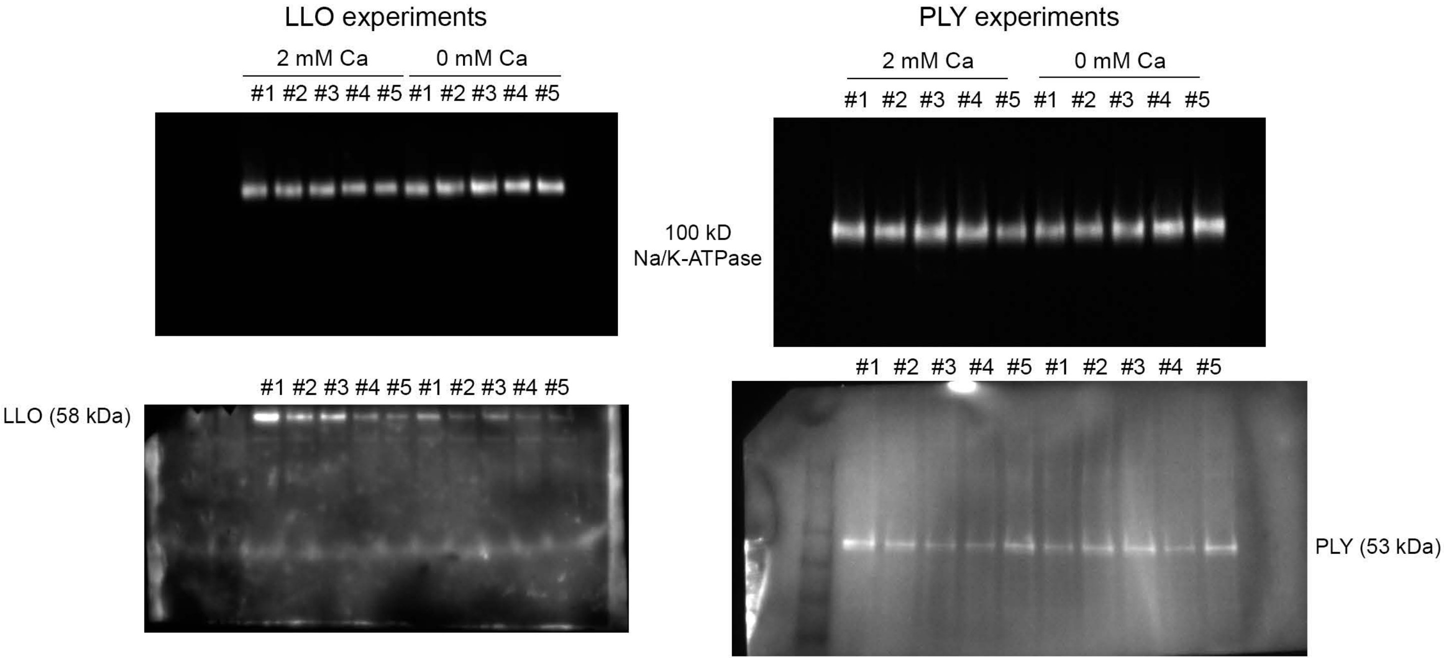

Supplementary figure S3. Unprocessed Western blot film scans of LLO experiments in Figure 4C including actin loading controls.

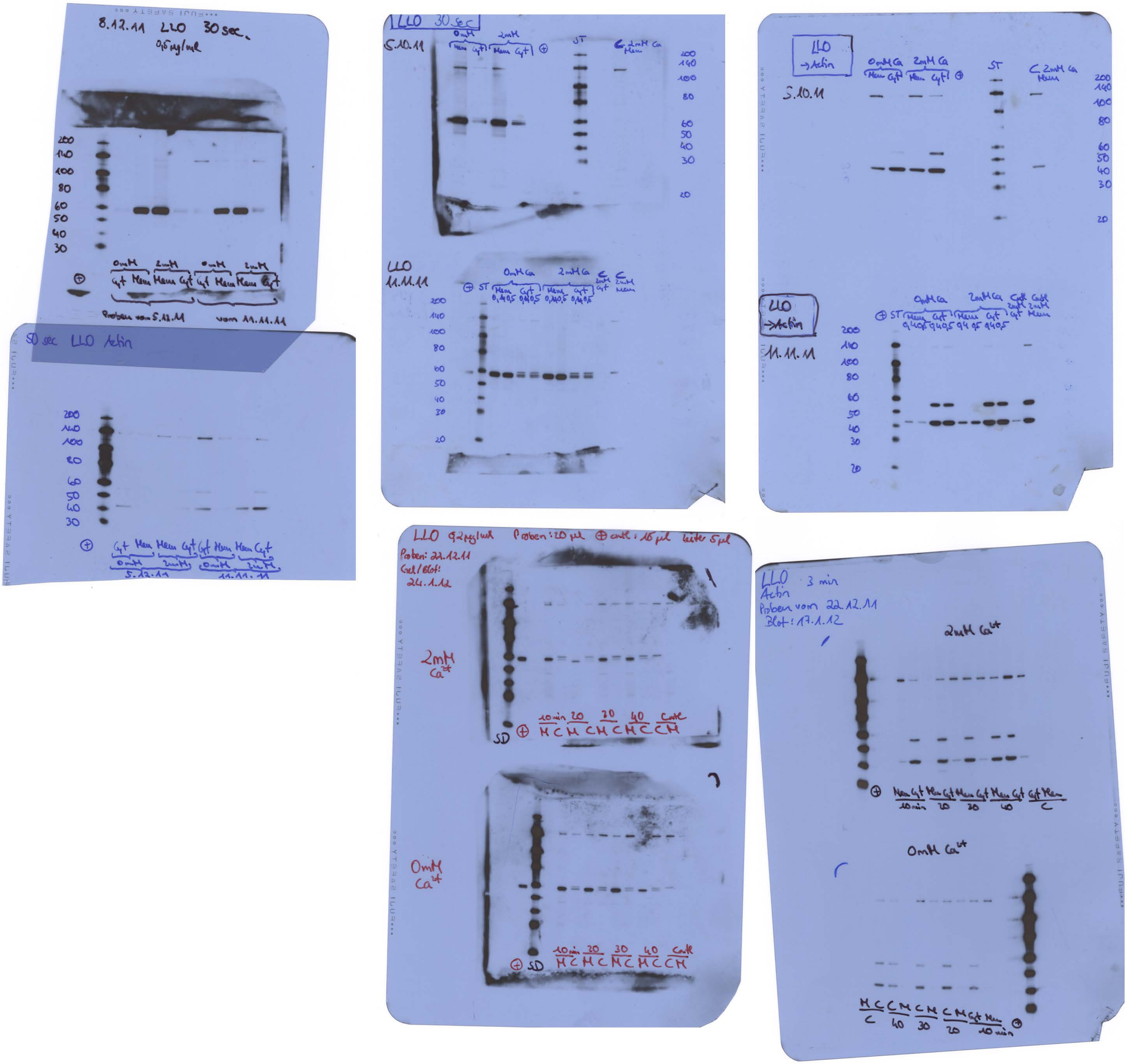

Supplementary figure S4. Unprocessed Western blot film scans of PLY experiments in Figure 4C including actin loading controls.

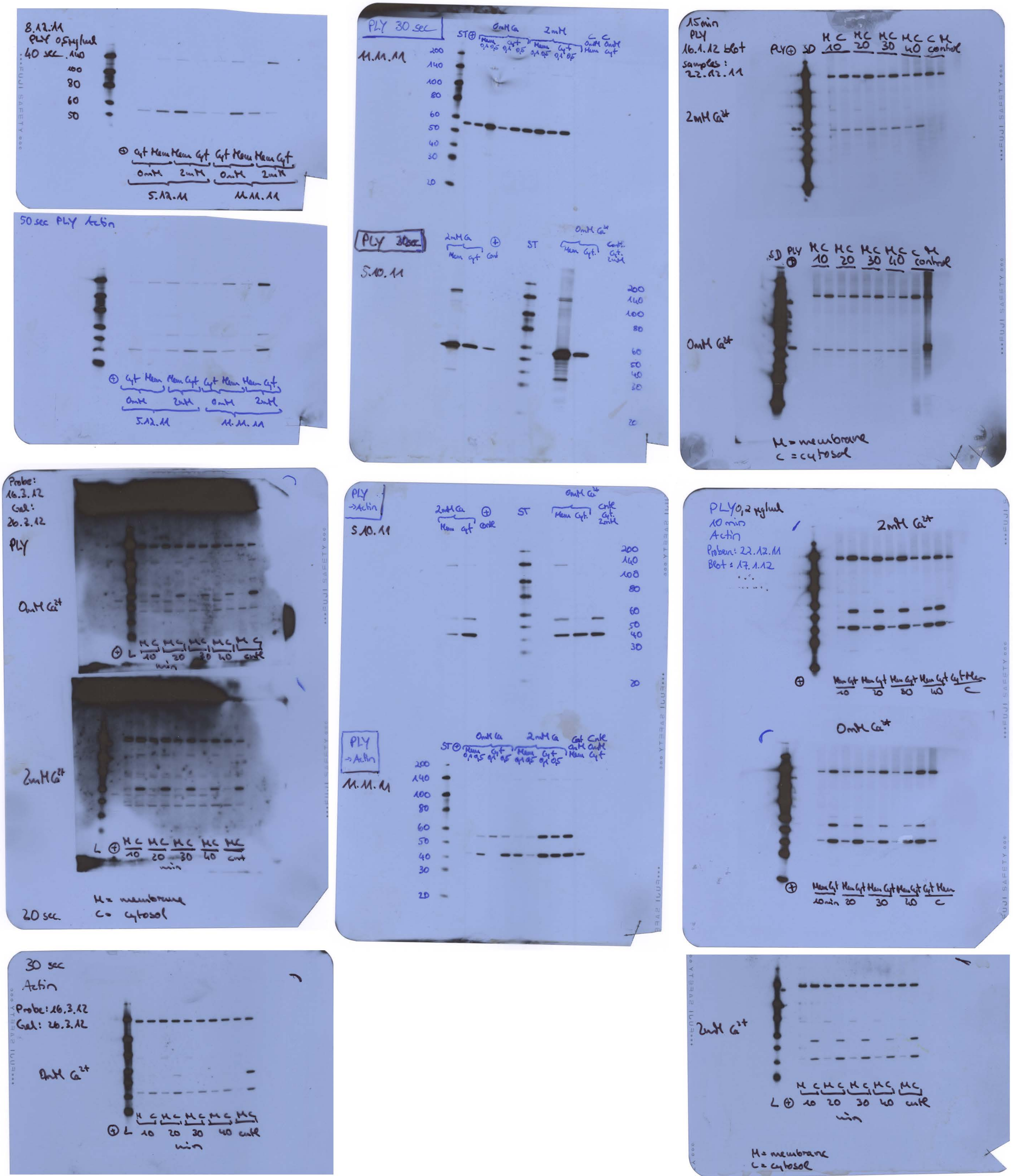

Supplement: Supplementary file 5 — Supplementary figures [file 41598_2018_34031_MOESM5_ESM.pdf]
